# Dendritic cell subsets dynamics and cytokine production in SIVmac239-infected Chinese rhesus macaques

**DOI:** 10.1186/1742-4690-7-102

**Published:** 2010-12-01

**Authors:** Hou-Jun Xia, Gao-Hong Zhang, Jian-Ping Ma, Zheng-Xi Dai, Shao-You Li, Jian-Bao Han, Yong-Tang Zheng

**Affiliations:** 1 Key Laboratory of Animal Models and Human Disease Mechanisms of Chinese Academy of Sciences and Yunnan province, Kunming Institute of Zoology, Chinese Academy of Sciences, Kunming, Yunnan 650223, China; 2 Graduate School of the Chinese Academy of Sciences, Beijing 100039, China

## Abstract

**Background:**

Several studies have demonstrated that SIV infection progresses more slowly to experimental AIDS in Chinese rhesus macaques (Ch Rhs) than in Indian rhesus macaques (Ind Rhs). Here we investigated the dynamic and functional changes in dendritic cell (DC) subsets in SIVmac239-infected Ch Rhs.

**Results:**

The numbers of both mDC and pDC strongly fluctuated but were not significantly changed during the acute and chronic phases of infection. However, the concentration of both poly (I:C)-induced IL-12 and HSV-1-induced IFN-α significantly increased in the acute phase of infection but returned to normal levels at the chronic phase of infection. The peak of IFN-α emerged earlier than that of IL-12, and it had a significantly positive correlation with IL-12, which indicated that IFN-α may initiate the immune activation. We also found that only the concentration of IFN-α was positively correlated with CD4+ T-cell counts, but it was negatively correlated with viral load.

**Conclusion:**

High levels of IFN-α in the early stage of infection may contribute to effective control of virus replication, and normal levels of IFN-α during chronic infection may help Ch Rhs resist the disease progression. The change in DC subsets dynamics and cytokine production may help further our understanding of why Ch Rhs are able to live longer without progressing to an AIDS-like illness.

## Background

Dendritic cells (DC) are a heterogeneous population of APC, essential in linking the innate and acquired immune response [1]. Two major DC subsets, CD11c+ myeloid DC (mDC) and CD123+ plasmacytoid DC (pDC), have been described in human [2] and non-human primates [3]. mDC play an important role in the acquired immune response by acquiring and processing viral antigens into peptides for major histocompatibility complex (MHC) presentation to T cells in secondary lymphoid organs [4]. As one of the DC precursors, pDC are located in blood and secondary lymphoid organs. They are specialized in rapidly secreting massive amounts of type 1 IFN following different viral (HIV, HSV-1) stimulations [5]. Then virus-activated pDC differentiate into a unique type of mature DC, which probably play a role in the initiation of the T-cell response in a manner similar to that of mDC [6]. Initially, mDC and pDC were thought to prime primarily type 1 and type 2 T-cell responses, respectively [7]. However, subsequent data suggested that pDC activated by influenza virus and CD40L are capable of priming type 1 response in an IL-12 and IFN-α-dependent fashion [8]. Type 1 responses are very important for controlling viral infections such as HIV.

DC are considered the first immune cells to encounter HIV and are involved in every stage of HIV infection. *In vitro*, both mDC and pDC are susceptible to infection by R5 and X4 HIV-1 isolates, although mDC are more efficiently infected by R5 HIV-1 [9]. Meanwhile, DCs may act as reservoirs for hiding HIV-1 and may then transmit HIV-1 to CD4 T-cells after DC migration into the lymph node [10,11]. *In vivo*, several studies have shown that both DC subsets are significantly reduced in HIV-infected patients' blood [12-17], with the decline being inversely correlated with viral load and reduced CD4^+ ^T-cell numbers [13,14]. This might be relative to the hypothesis that apoptosis of DC induced by HIV and/or migration of mature DC into the lymph node. The function of DC was impaired accompanying with the decline of cell number. Both mDC and pDC were severely impaired in their ability to stimulate T-lymphocyte proliferation in HIV-infected patients [18]. The IFN-α production of pDC with viral stimulation was also decreased in AIDS patients [15,19]. In addition to an IFN-α production deficit, antigen-presenting cells (APC) from HIV-infected subjects had reduced IL-12 production [20]. However, most studies in humans have been limited to the chronic stage of HIV infection, and animal models have mostly been used to investigate the early stage of infection.

The immune systems of non-human primates (NHP) closely resemble those of humans. The similar results were also observed that mDC and pDC were lost from the blood of SIV-infected Indian rhesus macaques (Ind Rhs) [21]. Chinese rhesus macaques (Ch Rhs) have recently been used in AIDS research as substitutes for their Indian counterparts. Compared with Ind Rhs, the SIVmac pathogenesis in Ch Rhs is closer to HIV-1 infection in untreated adult humans [22]. More and more reports have demonstrated that pDC could influence the disease progression by secreting IFN-α, so we suspect that DC subsets may be the main cause of the difference in progression to AIDS between Ch and Ind Rhs.

Here, we investigated the dynamics and function of blood DC subsets during acute and chronic SIVmac239 infection of Ch Rhs. We found that the numbers of mDC and pDC fluctuated strongly but were not significantly changed after SIVmac239 infection. The concentration of IL-12 and IFN-α significantly increased at the acute phase of infection, but remained at a normal level at the chronic phase of infection. The trends of change were more likely with African green monkeys, but not with Ind Rhs. This difference in change may be important in determining the AIDS progression.

## Results

### Virological outcome and CD4+ T-cell counts in challenged macaques

**Figure 1:**
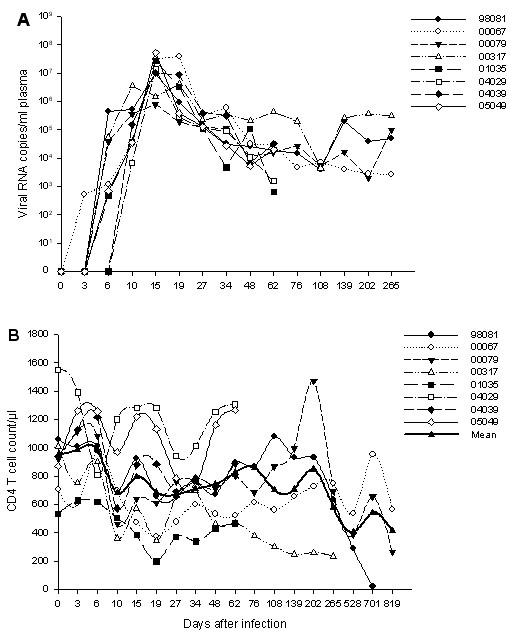
**The dynamics of viral load and CD4+ T-cell counts in Ch Rhs after SIVmac239 infection**. (A) Plasma viral load in SIVmac239-infected Ch Rhs; (B) Blood CD4+ T-cell counts during the infection.

The dynamics of viral load were investigated for each Ch Rhs. Each specimen sampled at different time points after infection was tested, and the samples spanned the acute and chronic phases of infection. Inoculation of rhesus macaques with SIVmac239 resulted in a high viraemia peak at day 14 post-infection (p.i) and a gradual persistent decline, with no animals studied completely controlling virus replication. The dynamics of viral load in the eight Ch Rhs are presented in Figure 1A. A different picture was observed for the two other Ch Rhs, 98081 and 00317, whose virus load increased significantly at the post-chronic phases and who died early p.i. because of AIDS.

The mean count of blood CD4+ T cells significantly decreased on days 10~34 p.i (*P *≤ 0.035), except on day 15. These returned to baseline level on days 48 to 202. They then decreased again on days 265 and 568 (*P *≤ 0.042), followed by a slower recovery (Figure 1B).

### The numbers of mDC and pDC showed no significant change during SIVmac239 infection

**Figure 2:**
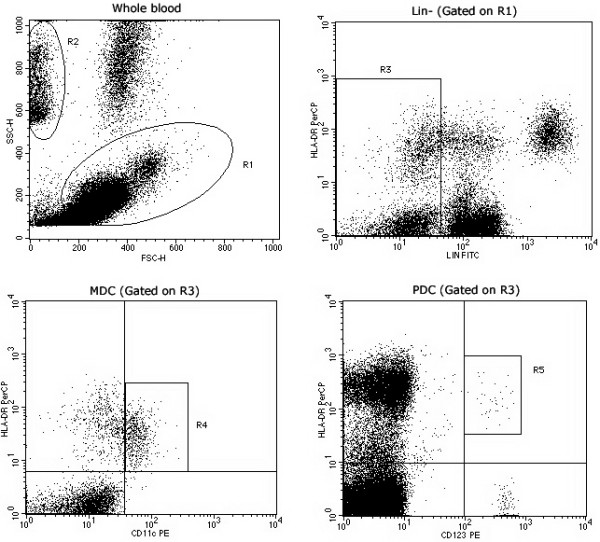
**Flow cytometric analysis of Ch Rhs mDC and pDC**. PBMC (R1) and TruCount beads (R2) were first gated on a forward-scatter/side-scatter (FSC/SSC) scattergram. Then Lin^- ^cells (CD3^-^CD14^-^CD20^-^) were selected in the R3 region. CD11c^+^HLA-DR^+ ^and CD123^+ ^HLA-DR^+ ^dots were gated in R4 and R5, respectively, from R1 × R3 gates.

For quantification of mDC and pDC, peripheral blood mononuclear cells (PBMC) (R1, Figure 2) and TruCount beads (R2, Figure 2) were first gated appropriately in the forward-scatter/side-scatter (FSC/SSC) scattergram using SSC as threshold. Lineage negative cells (R3, Figure 2) were gated from PBMC; HLA-DR^+^CD11c^+ ^of these cells were mDC (R4, Figure 2), while HLA-DR^+^CD123^+ ^were pDC (R5, Figure 2) [21,23]. The absolute cell count was calculated as follows: Cells concentration = (events in cells region×total number of beads in TruCount tube)/(events in beads region×sample volume).

**Figure 3:**
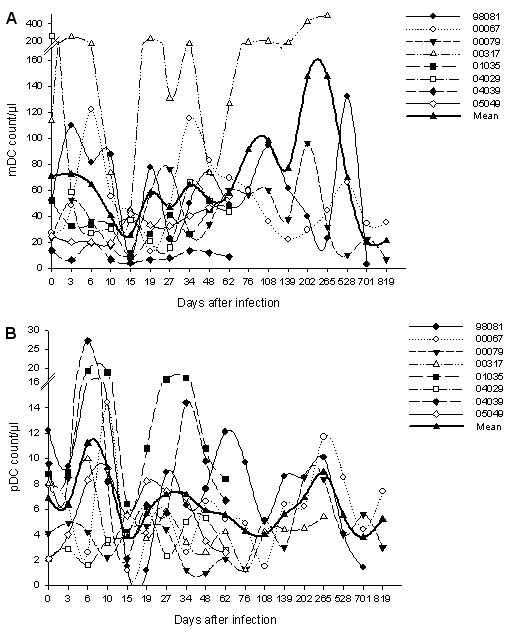
**The dynamics of mDC counts and pDC counts in Ch Rhs after SIVmac239 infection**. (A) Blood mDC counts during the infection; (B) Blood pDC counts during the infection.

The mean count of mDC before infection in the eight Ch Rhs was 70.7 cells/μl (range, 13.7 to 260.1 cells/μl); this fell to 25.0 cells/μl (range, 4 to 44.5 cells/μl) on day 15 p.i with an acute decrease, which may be explained by the migration of DC into the lymph node. Then the value gradually returned to the subnormal baseline level before day 76 and increased. Finally, the number declined and remained at a low level because of the death of 00317 and 98081 (Figure 3A). The mean count of mDC reached the highest site on days 202 and 265, but the value was not significantly increased (*P *= 0.144 and *P *= 0.273, respectively) because the higher count level of 00317 elevated the mean value. mDC did not show a significant increase or decrease during this period.

The change in pDC counts was different from that in mDC at the early stage of infection. The mean count of pDC showed an increase on day 6 p.i from 6.8 cells/μl (range, 2.1 to 12.2 cells/μl) to 11.1 cells/μl (range, 1.6 to 27.2 cells/μl) but sharply decreased to the nadir on day 15 p.i (mean, 3.8 cells/μl; range, 1.2 to 6.4 cells/μl). It returned to the subnormal level during the following four weeks and reached its second nadir on day 108 p.i (mean, 3.9 cells/μl; range, 1.5 to 5.1 cells/μl). The level of pDC increased again and fluctuated around baseline at the late stage of infection (Figure 3B).

### IL-12 and IFN-α produced by mDC and pDC, respectively, increased during acute SIVmac239 infection

**Figure 4:**
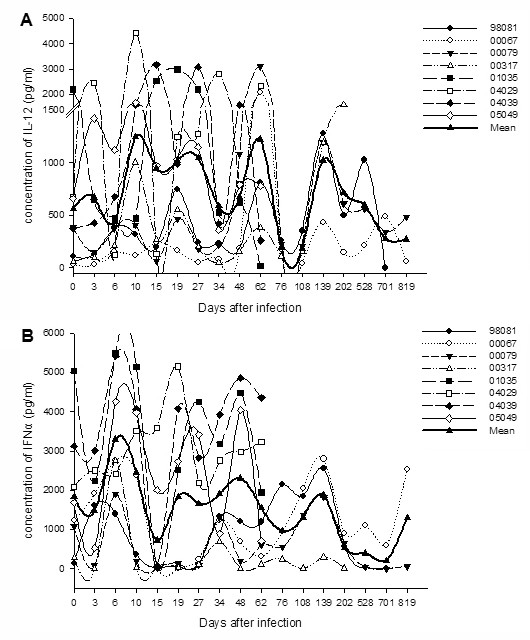
**The production of poly(I:C)-induced IL-12 or HSV-1-induced IFN-α *in vitro***. **(**A) IL-12 concentration in supernatants of Ch Rhs PBMC stimulated with poly(I:C); (B) IFN-α concentration in supernatants of Ch Rhs PBMC stimulated with HSV-1.

To determine whether the mDC and pDC functions were impaired, the IL-12 or IFN-α concentrations in the supernatants were investigated using TLR3L poly(I:C)- or TLR9L HSV-1-stimulated simian PBMC, respectively. As shown in Figure 4A, the mean amount of poly(I:C)-induced IL-12 was significantly increased on day 19 p.i (mean, 1021.7 pg/ml; range, 164 to 2984 pg/ml; *P *= 0.012) compared with that on pre-infection (mean, 562.3 pg/ml; range, 34 to 2194 pg/ml).

The concentrations of HSV-1-induced IFN-α also appeared transiently increased on day 6 p.i (mean, 3298 pg/ml; range, 1398 to 5499 pg/ml; *P *= 0.012), which was two weeks earlier than the increase in IL-12. They decreased after a week on day 15 p.i (mean, 728.3 pg/ml; range, 13 to 3591 pg/ml), and then recovered and remained at a subnormal level until monkeys 98081 and 00079 lost the ability for IFN-α production and died of AIDS (mean, 201 pg/ml; range, 0 to 604 pg/ml) (Figure 4B).

The increase in IL-12 and IFN-α during acute SIV infection could induce strong immune activation, which was considered to initiate AIDS progression in macaques [24]. However, these cytokines, especially IFN-α, are necessary to inhibit disease progression during chronic infection. Noticeably, the trend in IFN-α secretion in monkey 00317 was different from that in other monkeys. The pDC of 00317 were weak in secreting IFN-α pre-infection, but they were able to secret a high level of IFN-α on day 6 p.i. They could not release an abundance of IFN-α after day 48 and just kept to a subnormal level of the baseline. The low concentrations of IFN-α in the post-chronic phase of infection may have led to a quick death.

### Enhanced IL-12 production per mDC and reduced IFN-α production per pDC during acute SIVmac239 infection

**Figure 5:**
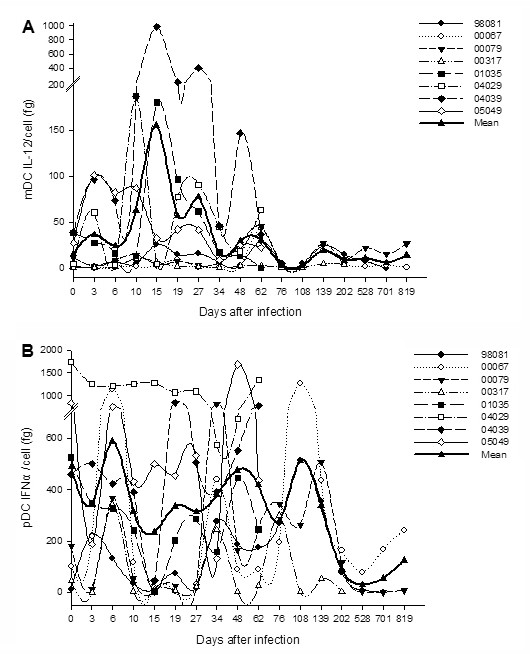
**The production of poly(I:C)-induced IL-12 or HSV-1-induced IFN-α per individual mDC or pDC *in vitro***. **(**A) IL-12 production per mDC in Ch Rhs; (B) IFN-α production per pDC in Ch Rhs.

At baseline, the mean amount of IL-12 produced by a single mDC was 15.7 fg (range, 0.7 to 39.0 fg), and that of IFN-α produced by a single pDC was 489.1 fg (range, 12 to 1745 fg). The baseline value of IL-12 was half lower than that in humans (34.7 fg). After infection, IL-12 production per mDC was significantly increased from days 19 (mean, 57 fg; range, 2.0 to 205.8 fg; *P *= 0.025) to 27 (mean, 77.6 fg; range, 0.8 to 405 fg; *P *= 0.043) p.i. Afterward, the levels of IL-12 were gradually decreased to a subnormal level (Figure 5A). The baseline value of IFN-α produced by a single pDC was seven-fold higher than that in humans (65.8 fg). The mean amount of IFN-α significantly decreased on days 10 (mean, 316.3 fg; range, 5.0 to 1251.1 fg; *P *= 0.036), 15 (mean, 234.4 fg; range, 2.3 to 1282.5 fg; *P *= 0.012), and 27 (mean, 314.4 fg; range, 17.5 to 1091.5 fg; *P *= 0.05) p.i. The IFN-α production was recovered following and then reduced at day 528 (mean, 30.1 fg; range, 4.6 to 76.9 fg) p.i (Figure 5B). SIV infection significantly influenced the cytokine-releasing capacity of DC subsets.

### Only IFN-α is positively correlated with CD4+ T-cell counts but is negatively correlated with viral load through the infection

**Figure 6:**
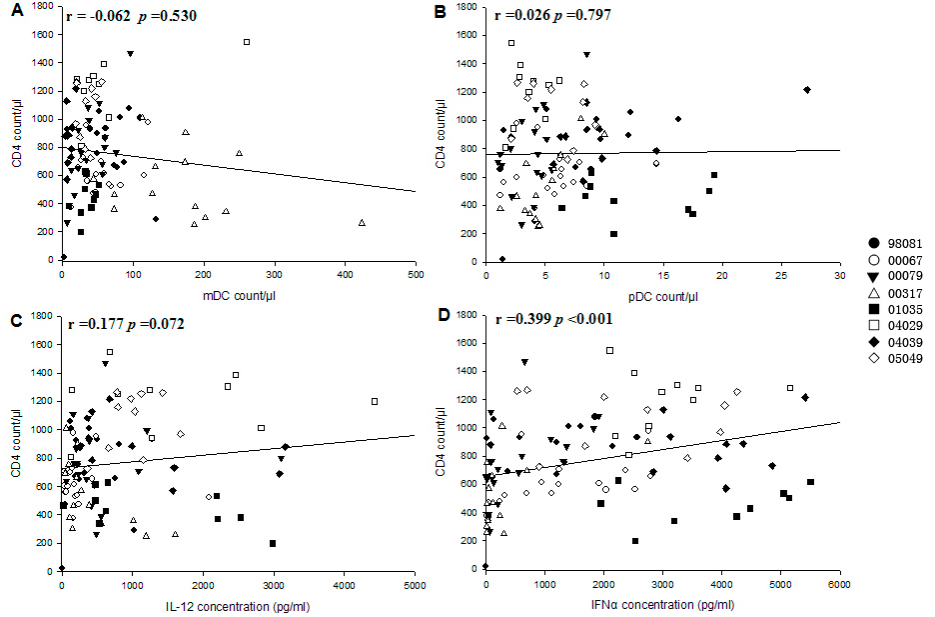
**The analysis of correlations between CD4+ T-cell counts and DC subset counts or cytokine production**. (A) CD4+ T-cell counts and mDC counts; (B) CD4+ T-cell counts and pDC counts; (C) CD4+ T-cell counts and IL-12 concentration; (D) CD4+ T-cell counts and IFN-α concentration.

There was no statistical correlation between the absolute numbers of mDC with CD4+ T cells counts (Figure 6A) during infection, nor was this correlated with pDC (Figure 6B). However, the pDC counts were positively correlated with the CD4+ T-cell counts (r = 0.479, *P *= 0.018) during the chronic phase of infection (76 to 819 p.i), as previously reported [25]. A positive correlation was observed between the concentrations of IFN-α and the CD4+ T-cell counts (Figure 6D; r = 0.399, *P *< 0.001), while no correlation was found between the concentrations of IL-12 and the CD4+ T-cell counts (Figure 6C).

**Figure 7:**
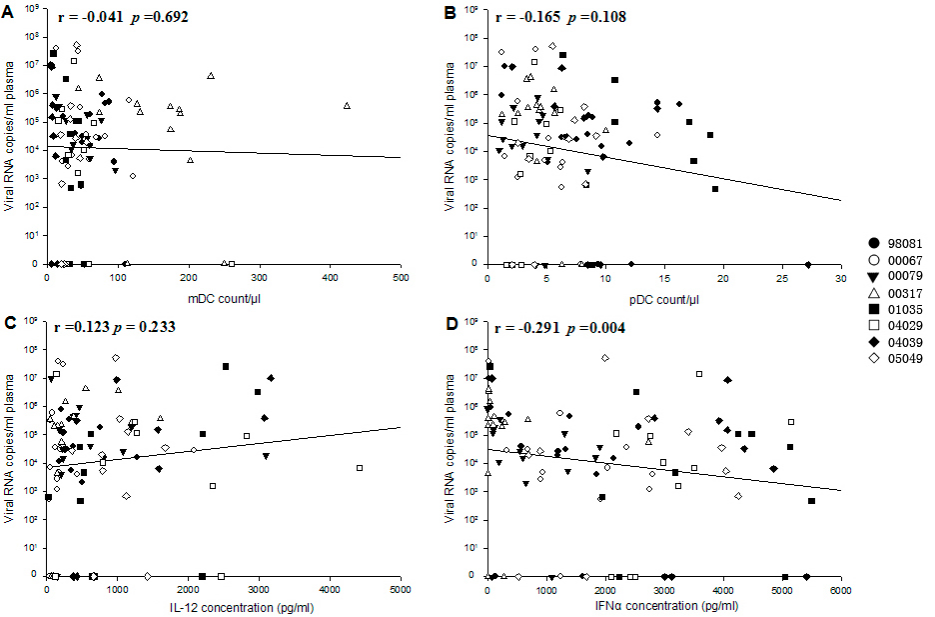
**The analysis of correlations between plasma viral load and DC subset counts or cytokine production**. (A) plasma viral load and mDC counts; (B) plasma viral load and pDC counts; (C) plasma viral load and IL-12 concentration; (D) plasma viral load and IFN-α concentration.

Both the counts of DC subsets (Figure 7A, B) and the concentrations of IL-12 (Figure 7C) were not correlated with viral load. Only the concentrations of IFN-α were negatively correlated with viral load (Figure 7D; r = -0.291, *P *= 0.004). In addition, the concentrations of IFN-α were also negatively correlated with the mDC counts (r = -0.268, *P *= 0.006)and positively correlated with the pDC counts (r = 0.454, *P *< 0.001) and IL-12 concentrations (r = 0.311, *P *= 0.001). Our results showed that IFN-α may be a good choice for predicting disease progression.

## Discussion

In this study, we detected the dynamic and functional changes in mDC and pDC in SIVmac239-infected Ch Rhs. Both the mDC and pDC numbers were observed to decrease on day 15 p.i, while the virus load achieved culmination on that day. This decrease may be due to the migration of DC into the lymph node [26,27]. mDC returned to normal levels and showed no significant increase or decrease. pDC increased within the first week, which was also described using SIVmac251-infected Ind Rhs [28]. This transient increase was also observed in SIVmac251-infected young adult cynomolgus macaques (*Macaca fascicularis*) [29], but not in SIVagm-infected African green monkeys (*Chlorocebus sabaeus*) [25]. The results suggest pDC were sensitive to the invading virus and accumulated in the blood after intravenous virus challenge. Different changes in pDC were observed between Ch and Ind Rhs after the first nadir. In SIV-infected Ch Rhs, the pDC numbers were recovered during the chronic phase of infection, while no appreciable recovery was observed in their Indian counterparts [30]. As previously reported, Ch Rhs has relatively slow progression to AIDS compared with Ind Rhs [22]. The pDC trend in Ch Rhs was more similar to that in African green monkeys [25] which are natural hosts for SIVagm and generally do not progress to AIDS, despite having high levels of plasma viral load.

Most studies have found that blood mDC and pDC are severely depleted in HIV-infected patients [12-17] or rhesus macaques with AIDS [21]. However, Soumelis et al. [17] also showed there were high levels of pDC in long-term survivors who had been infected for more than 10 years with no clinical sign of disease. This suggests that an increased pDC number may help protect against disease development. The numbers and percentages of mDC and pDC were not observed to be significantly changed in this study, and they fluctuated around the baseline level after SIVmac239 infection. It seems that Ch Rhs avoid the rapid depletion of the DC subsets, and they may mount more potent immune responses to SIVmac239 [31]. The numbers of both mDC and pDC had no significant relationship with CD4+ T cells, as in other reports [30]. However, the pDC counts were positively correlated with the CD4+ T-cell counts during the chronic phase of SIV infection. The recovery of pDC during the chronic phase of infection plays an important role in prolonging the progress of SIV-infected Ch Rhs toward AIDS.

Impaired DC functions may play important roles in the immune deficiencies of SIVmac infection. The cytokine-releasing capacity is a pivotal function of DC in resisting virus infection. Circulating mDC preferentially express TLR3 and exclusively secrete IL-12 stimulated by poly(I:C) [32,33]. Stimulation by HSV-1 generally occurs through TLR9, which is strongly expressed by pDC. Several papers have demonstrated that pDC are the major producers of IFN-α when HSV-1 directly stimulates the human or simian PBMC [6,34]. We examined the change in cytokine secretion of DC subsets during SIV infection. The ability of mDC to secrete IL-12 after viral infection has been investigated extensively because this molecule is crucial to inducing Th1-skewed antiviral responses *in vivo *[35]. In our study, the amount of IL-12 significantly increased on day 19 p.i and remained at a high level at most time points. The increase was also observed by Byrnes et al. [36], who found that patients with acute/early HIV infection exhibited *in vivo *IL-12 production along with increased maximal IL-12 production by their PBMC *in vitro *in the absence and presence of HAART. This increase showed significant immune activation at the acute/early phase of HIV/SIV infection. However, keeping IL-12 at a high level in the chronic stage is opposed to the finding in previous research that the concentration of IL-12 produced by mDC was significantly reduced in chronically HIV-infected patients [32,37].

The IFN-α mainly secreted by pDC exerts a strong anti-HIV activity not only directly, but also indirectly through the activation of the immune system. During SIVmac239 infection, the concentration of IFN-α stimulated simian PBMC with HSV-1 significantly increased within the first week, and kept normal levels later. This transitory peak of IFN-α was previously found in African green monkeys using the same method [25]. IFN-α has proven to be beneficial in controlling HIV replication during the early stages of infection [38]. Our results indicated that pDC were motivated quickly to eliminate SIV after infection in Ch Rhs. High-level IFN-α was hard to control in rhesus macaques [39], which could cause chronic immune activation, and finally lead to CD4+ T-cell depletion and AIDS progression [40]. Nevertheless, low-level IFN-α was useless in controlling the disease progression during chronic infection. It has long been known that there is a defect in IFN-α production by PBMC or pDC in chronically HIV-infected patients [15,19,37,41,42] and that IFN-α production is higher in asymptomatic long-term survivors than in uninfected controls [17]. The rebound of IFN-α production during chronic infection may help Ch Rhs resist the disease progression. Although there is a lack of direct evidence for the IFN-α change in chronically SIV-infected Ind Rhs, the persistent low counts and percentages of pDC could predict a low concentration of IFN-α. Compared with African green monkeys, Ch Rhs had a similar IFN-α change after SIV infection and had slow progression toward AIDS. Our results also showed that the concentration of IFN-α was significantly positively correlated with the CD4+ T-cell counts but negatively correlated with viral load through the infection. Thus, it can be used to predict AIDS progression.

When we excluded the impact of the number, we found that the change in IFN-α production per pDC was not statistically significant on day 6 p.i. Thus, the increase in total IFN-α production was mainly determined by the number of pDC, which significantly decreased on days 10, 15, and 27, and then returned to a normal level. This result is inconsistent with Malleret et al. [29] who reported that IFN-I production per pDC of cynomolgus macaques was significantly lower in response to HSV-1 on day 35 after infection and recovered 9 months after infection. However, this decline does not mean that the pDC production capacity of IFN-α has been impaired, since pDC retained largely normal functions in response to TLR7 stimulation during acute SIV infection, as found using a flow cytometric assay to detect IFN-α-producing cells [28]. Human pDC rapidly become refractory to secondary stimulation [43], which was considered as the cause of the decrease in IFN-α production *in vitro *in patients infected with HIV [44]. Thus, the decrease in IFN-α production per pDC in our study demonstrated that pDC were quickly refractory to IFN-α production in response to *de novo *stimulation. Indeed, we have also detected a transient peak in IFN-α concentration in plasma in the acute phase of infection (data not shown), as previously found in SIV-infected Ind Rhs [45] and African green monkeys [25] at around day 10 p.i. This peak in IFN-α in plasma resulted from the intense stimulation of pDC by the high plasma viral load *in vivo*.

In contrast with IFN-α, IL-12 production per mDC increased between days 10 and 27 p.i, and had no significant change in the following days of SIVmac239 infection in our study. Increased IL-12 production per mDC was closely following with the increase of IFN-α concentration in plasma, and the peak of IFN-α level in plasma was accordance with that of increased-IL-12. There was a significant positive correlation between the concentration of IFN-α and that of IL-12, but a negative correlation was seen between the concentration of IFN-α and mDC counts. HIV-activated pDC were able to induce the bystander maturation of mDC through IFN-α [46]. Thus, our results indicated that IFN-α might prompt mDC to secrete more IL-12 after maturation. It is a protective strategy of IFN-α to recruit more immune cells, like mDC, to defend against HIV infection.

## Conclusions

In summary, our study revealed that the counts of mDC and pDC did not significantly change during SIVmac239 infection in Ch Rhs and had no relationship with CD4+ T cells or viral load. Poly(I:C)-induced IL-12 and HSV-1-induced IFN-α significantly increased at the acute phase of infection, but returned to normal levels thereafter. The concentration of IFN-α showed a significantly positive correlation with the CD4+ T-cell counts, but had a negative correlation with viral load. High levels of IFN-α in the early stage of infection contribute to the effective control of virus replication and also initiated the AIDS progression, while median levels of IFN-α concentration during chronic infection may help Ch Rhs resist the AIDS progression. The dynamics of IFN-α secreted by pDC might be the main cause of the slow progression to AIDS in SIV-infected Ch Rhs.

## Materials and methods

### Animals and infections

The eight Ch Rhs (*Macaca mulatta*) used in this study were from the Kunming Primate Research Center, Chinese Academy of Sciences (CAS), and housed at the ABSL-3 laboratory in accordance with the Guide for the Committee on Animals for KIZ, CAS, and the Animal Welfare Act. The Ch Rhs were adult males between 5 and 11 years old; each weighed 6 to 12 kg. All eight monkeys were negative for SIV, STLV, and SRV when included in the study, as demonstrated by enzyme-linked immunosorbent assay or PCR analyses.

Rhesus macaques were inoculated intravenously with 5 × 10^3 ^50% tissue culture infectious doses of SIVmac239. Four of these (00067, 00079, 00317, and 98081) were observed for 819 days post-inoculation or until death from AIDS. Two of the macaques, 00317 and 98081, progressed to AIDS and died at 69 and 94 weeks post-inoculation, respectively. Four others (05049, 04029, 04039, and 01035) were observed only during the early phase of infection (62 days p.i).

### Quantification of viruses in plasma

The levels of viral RNA in plasma were measured by an in-house real-time PCR method. Briefly, plasma was separated from whole blood collected in EDTA-K_2_-containing tubes. Viral RNA was extracted using the High Pure Viral RNA Kit (Roche) according to the manufacturer's instructions. The samples were analyzed immediately for real-time PCR or stored at -80°C until use. A two-step RT-qPCR assay using the PrimeScript™ RT reagent Kit and Premix Ex Taq™ (Takara) was performed on a 7500 Fast Real-Time PCR System (Applied Biosystems). PCR reactions in a total volume of 20 μl consisted of 10 μl of Premix Ex Taq, 2 μl of the standard or samples, 0.4 μl ROX reference Dye II, 200 nM of each primer, and 100 nM of the TaqMan probe. The probe and primers were designed to bind within the conserved SIVmac gag region. The sequences of the primers used were: 5'-TCGGTCTTAGCTCCATTAGTGCC-3' and 5'-GCTTCCTCAGTGTGTTTCACTTTC-3'; the TaqMan probe sequence was: 5' -CTTCTGCGTGAATGCACCAGATGACGC-3'. In the probe the fluorescence reporter dye at the 5' end was FAM (6-carboxyfluorescein), and the quencher dye at the 3' end was TAMRA (6-carboxytetramethylrhodamine). The control template is an *in vitro *transcript pGEM-4Z-SIVgag357containing the SIV gag fragment from SIVmac239, prepared from the plasmid p239SpSp5' kindly provided by Dr. Bin Gao (Institute of Child Health, University College London, UK). RNA transcripts were diluted in nuclease-free water and stored at -80°C in single-use aliquots. For each run, an RNA standard curve was generated in *in vitro *transcripts ranging from 6~6 × 10^6 ^to the nominal copy equivalents/reaction. One thousand copies per ml were considered the limit of detection.

### Flow cytometry

Flow cytometric analyses were performed using whole blood. The number of T-cell subsets, mDC, and pDC in peripheral blood was determined using the true-count method, as previously described [23]. For identification of DC subsets, whole blood was incubated in BD TruCount tubes with a lineage (Lin) mixture of FITC-conjugated mAb against CD3 (clone SP34; BD), CD14 (clone TÜK4; Miltenyi), and CD20 (clone LT20; Miltenyi), with HLA-DR-PerCP (clone L243; BD). Then CD11c-PE (clone 3.9; eBioscience), CD123-PE (clone 7G3; BD), or isotype mAbs were added separately into each tube and incubated for 15 min at RT. FACS lysing solution (BD Biosciences) was added into each tube to lyse erythrocyte and fix samples. Around 30,000 cells were acquired with three-color flow cytometry using FACSCalibur (Becton Dickinson) and then analyzed through CellQuest software.

### PBMC isolation and *in vitro *stimulation

PBMC were isolated by Ficoll-Paque (GE Healthcare) density gradient centrifugation. PBMC were cultured in 24-well plates (Costar) at 4 × 10^6 ^cells/ml in RPMI 1640 supplemented with 10% fetal bovine serum. A total of 25 μg/ml poly(I:C) or HSV-1 at a MOI of 1.0 was added to stimulate mDC or pDC, respectively. After 24 hours of culture, cell-free supernatants were harvested and kept at -80°C until assayed.

### Cytokine assays

Samples of culture supernatants were analyzed for IL-12 (p40+p70) and IFN-α using a commercially available ELISA^pro ^kit for human IL-12 (total) (Mabtech) and a multi-subtype human IFN-α ELISA kit (PBL Biomedical Laboratories) according to the manufacturer's instructions. Per cell IL-12 or IFN-α production was calculated using the formula described by Zhang et al. [32] as IL-12/(mDC% × 4 × 10^6^) and IFN-α/(pDC% × 4 × 10^6^).

### Statistical analysis

All data were analyzed using the SPSS 13.0 software. The nonparametric Wilcoxon rank test was used to compare data from the same macaque at different time points before and after SIV infection. The nonparametric Spearman rank correlation test was used to investigate the relationship between parameters. For all tests, two-sided *p *< 0.05 was considered to be significant.

## Competing interests

The authors declare that they have no competing interests.

## Authors' contributions

YTZ and HJX designed this study. HJX and GHZ carried out the experiments. JPM, ZXD, SYL, and JBH coordinated and performed the primate studies. YTZ, HJX, and GHZ analyzed the results and drafted the manuscript. All authors read and approved the final manuscript.

## References

[B1] Banchereau J Briere F Caux C Davoust J Lebecque S Liu YJ Pulendran B Palucka K Immunobiology of dendritic cells Annu Rev Immunol 2000 18 767 811 10.1146/annurev.immunol.18.1.767 10837075

[B2] Liu YJ Dendritic cell subsets and lineages, and their functions in innate and adaptive immunity Cell 2001 106 259 262 10.1016/S0092-8674(01)00456-1 11509173

[B3] Coates PT Barratt-Boyes SM Zhang L Donnenberg VS O'Connell PJ Logar AJ Duncan FJ Murphey-Corb M Donnenberg AD Morelli AE Maliszewski CR Thomson AW Dendritic cell subsets in blood and lymphoid tissue of rhesus monkeys and their mobilization with Flt3 ligand Blood 2003 102 2513 2521 10.1182/blood-2002-09-2929 12829599

[B4] Banchereau J Steinman RM Dendritic cells and the control of immunity Nature 1998 392 245 252 10.1038/32588 9521319

[B5] Siegal FP Kadowaki N Shodell M Fitzgerald-Bocarsly PA Shah K Ho S Antonenko S Liu YJ The nature of the principal type 1 interferon-producing cells in human blood Science 1999 284 1835 1837 10.1126/science.284.5421.1835 10364556

[B6] Liu YJ IPC: professional type 1 interferon-producing cells and plasmacytoid dendritic cell precursors Annu Rev Immunol 2005 23 275 306 10.1146/annurev.immunol.23.021704.115633 15771572

[B7] Rissoan MC Soumelis V Kadowaki N Grouard G Briere F de Waal Malefyt R Liu YJ Reciprocal control of T helper cell and dendritic cell differentiation Science 1999 283 1183 1186 10.1126/science.283.5405.1183 10024247

[B8] Cella M Facchetti F Lanzavecchia A Colonna M Plasmacytoid dendritic cells activated by influenza virus and CD40L drive a potent TH1 polarization Nat Immunol 2000 1 305 310 10.1038/79747 11017101

[B9] Smed-Sörensen A Loré K Vasudevan J Louder MK Andersson J Mascola JR Spetz AL Koup RA Differential susceptibility to human immunodeficiency virus type 1 infection of myeloid and plasmacytoid dendritic cells J Virol 2005 79 8861 8869 10.1128/JVI.79.14.8861-8869.2005 15994779 PMC1168781

[B10] Coleman CM Wu L HIV interactions with monocytes and dendritic cells: viral latency and reservoirs Retrovirology 2009 6 51 10.1186/1742-4690-6-51 19486514 PMC2697150

[B11] Thibault S Fromentin R Tardif MR Tremblay MJ TLR2 and TLR4 triggering exerts contrasting effects with regard to HIV-1 infection of human dendritic cells and subsequent virus transfer to CD4+ T cells Retrovirology 2009 6 42 10.1186/1742-4690-6-42 19419540 PMC2691729

[B12] Almeida M Cordero M Almeida J Orfao A Different subsets of peripheral blood dendritic cells show distinct phenotypic and functional abnormalities in HIV-1 infection AIDS 2005 19 261 271 15718836

[B13] Barron MA Blyveis N Palmer BE MaWhinney S Wilson CC Influence of plasma viremia on defects in number and immunophenotype of blood dendritic cell subsets in human immunodeficiency virus 1-infected individuals J Infect Dis 2003 187 26 37 10.1086/345957 12508143

[B14] Donaghy H Pozniak A Gazzard B Qazi N Gilmour J Gotch F Patterson S Loss of blood CD11c(+) myeloid and CD11c(-) plasmacytoid dendritic cells in patients with HIV-1 infection correlates with HIV-1 RNA virus load Blood 2001 98 2574 2576 10.1182/blood.V98.8.2574 11588058

[B15] Feldman S Stein D Amrute S Denny T Garcia Z Kloser P Sun Y Megjugorac N Fitzgerald-Bocarsly P Decreased interferon-alpha production in HIV-infected patients correlates with numerical and functional deficiencies in circulating type 2 dendritic cell precursors Clin Immunol 2001 101 201 210 10.1006/clim.2001.5111 11683579

[B16] Pacanowski J Kahi S Baillet M Lebon P Deveau C Goujard C Meyer L Oksenhendler E Sinet M Hosmalin A Reduced blood CD123+ (lymphoid) and CD11c+ (myeloid) dendritic cell numbers in primary HIV-1 infection Blood 2001 98 3016 3021 10.1182/blood.V98.10.3016 11698285

[B17] Soumelis V Scott I Gheyas F Bouhour D Cozon G Cotte L Huang L JA and Liu YJ Depletion of circulating natural type 1 interferon-producing cells in HIV-infected AIDS patients Blood 2001 98 906 912 10.1182/blood.V98.4.906 11493432

[B18] Donaghy H Gazzard B Gotch F Patterson S Dysfunction and infection of freshly isolated blood myeloid and plasmacytoid dendritic cells in patients infected with HIV-1 Blood 2003 101 4505 4511 10.1182/blood-2002-10-3189 12576311

[B19] Chehimi J Campbell DE Azzoni L Bacheller D Papasavvas E Jerandi G Mounzer K Kostman J Trinchieri G Montaner LJ Persistent decreases in blood plasmacytoid dendritic cell number and function despite effective highly active antiretroviral therapy and increased blood myeloid dendritic cells in HIV-infected individuals J Immunol 2002 168 4796 4801 11971031 10.4049/jimmunol.168.9.4796

[B20] Chougnet C Wynn TA Clerici M Landay AL Kessler HA Rusnak J Melcher GP Sher A Shearer GM Molecular analysis of decreased interleukin-12 production in persons infected with human immunodeficiency virus J Infect Dis 1996 174 46 53 8656012 10.1093/infdis/174.1.46

[B21] Brown KN Trichel A Barratt-Boyes SM Parallel loss of myeloid and plasmacytoid dendritic cells from blood and lymphoid tissue in simian AIDS J Immunol 2007 178 6958 6967 17513745 10.4049/jimmunol.178.11.6958

[B22] Ling B Veazey RS Luckay A Penedo C Xu K Lifson JD Marx PA SIV(mac) pathogenesis in rhesus macaques of Chinese and Indian origin compared with primary HIV infections in humans AIDS 2002 16 1489 1496 10.1097/00002030-200207260-00005 12131186

[B23] Xia HJ Zhang GH Wang RR Zheng YT The influence of age and sex on the cell counts of peripheral blood leukocyte subpopulations in Chinese rhesus macaques Cell Mol Immunol 2009 6 433 440 10.1038/cmi.2009.55 20003819 PMC4003037

[B24] Pandrea I Sodora DL Silvestri G Apetrei C Into the wild: simian immunodeficiency virus (SIV) infection in natural hosts Trends Immunol 2008 29 419 428 10.1016/j.it.2008.05.004 18676179 PMC2840226

[B25] Diop OM Ploquin MJ Mortara L Faye A Jacquelin B Kunkel D Lebon P Butor C Hosmalin A Barré-Sinoussi F Müller-Trutwin MC Plasmacytoid dendritic cell dynamics and alpha interferon production during Simian immunodeficiency virus infection with a nonpathogenic outcome J Virol 2008 82 5145 5152 10.1128/JVI.02433-07 18385227 PMC2395206

[B26] Loré K Sönnerborg A Broström C Goh LE Perrin L McDade H Stellbrink HJ Gazzard B Weber R Napolitano LA van Kooyk Y Andersson J Accumulation of DC-SIGN+CD40+ dendritic cells with reduced CD80 and CD86 expression in lymphoid tissue during acute HIV-1 infection AIDS 2002 16 683 692 10.1097/00002030-200203290-00003 11964524

[B27] Zimmer MI Larregina AT Castillo CM Capuano S Falo LD Jr Murphey-Corb M Reinhart TA Barratt-Boyes SM Disrupted homeostasis of Langerhans cells and interdigitating dendritic cells in monkeys with AIDS Blood 2002 99 2859 2868 10.1182/blood.V99.8.2859 11929776

[B28] Brown KN Wijewardana V Liu X Barratt-Boyes SM Rapid influx and death of plasmacytoid dendritic cells in lymph nodes mediate depletion in acute simian immunodeficiency virus infection PLoS Pathog 2009 5 e1000413 10.1371/journal.ppat.1000413 19424421 PMC2671605

[B29] Malleret B Manéglier B Karlsson I Lebon P Nascimbeni M Perié L Brochard P Delache B Calvo J Andrieu T Spreux-Varoquaux O Hosmalin A Le Grand R Vaslin B Primary infection with simian immunodeficiency virus: plasmacytoid dendritic cell homing to lymph nodes, type I interferon, and immune suppression Blood 2008 112 4598 4608 10.1182/blood-2008-06-162651 18787223

[B30] Reeves RK Fultz PN Disparate effects of acute and chronic infection with SIVmac239 or SHIV-89.6P on macaque plasmacytoid dendritic cells Virology 2007 365 356 368 10.1016/j.virol.2007.03.055 17490699 PMC2043480

[B31] Monceaux V Viollet L Petit F Cumont MC Kaufmann GR Aubertin AM Hurtrel B Silvestri G Estaquier J CD4+ CCR5+ T-cell dynamics during simian immunodeficiency virus infection of Chinese rhesus macaques J Virol 2007 81 13865 13875 10.1128/JVI.00452-07 17898067 PMC2168866

[B32] Zhang Z Fu J Zhao Q He Y Jin L Zhang H Yao J Zhang L Wang FS Differential restoration of myeloid and plasmacytoid dendritic cells in HIV-1-infected children after treatment with highly active antiretroviral therapy J Immunol 2006 176 5644 5651 16622034 10.4049/jimmunol.176.9.5644

[B33] Kadowaki N Ho S Antonenko S Malefyt RW Kastelein RA Bazan F Liu YJ Subsets of human dendritic cell precursors express different toll-like receptors and respond to different microbial antigens J Exp Med 2001 194 863 869 10.1084/jem.194.6.863 11561001 PMC2195968

[B34] Chung E Amrute SB Abel K Gupta G Wang Y Miller CJ Fitzgerald-Bocarsly P Characterization of virus-responsive plasmacytoid dendritic cells in the rhesus macaque Clin Diagn Lab Immunol 2005 12 426 435 15753256 10.1128/CDLI.12.3.426-435.2005 PMC1065203

[B35] Trinchieri G Interleukin-12 and the regulation of innate resistance and adaptive immunity Nat Rev Immunol 2003 3 133 146 10.1038/nri1001 12563297

[B36] Byrnes AA Harris DM Atabani SF Sabundayo BP Langan SJ Margolick JB Karp CL Immune activation and IL-12 production during acute/early HIV infection in the absence and presence of highly active, antiretroviral therapy J Leukoc Biol 2008 84 1447 1453 10.1189/jlb.0708438 18806124 PMC2614601

[B37] Anthony DD Yonkers NL Post AB Asaad R Heinzel FP Lederman MM Lehmann PV Valdez H Selective impairments in dendritic cell-associated function distinguish hepatitis C virus and HIV infection J Immunol 2004 172 4907 4916 15067070 10.4049/jimmunol.172.8.4907

[B38] Lane HC Davey V Kovacs JA Feinberg J Metcalf JA Herpin B Walker R Deyton L Davey RT Falloon J Interferon-alpha in patients with asymptomatic human immunodeficiency virus (HIV) infection. A randomized, placebo-controlled trial Ann Intern Med 1990 112 805 811 1971503 10.7326/0003-4819-112-11-805

[B39] Jacquelin B Mayau V Targat B Liovat AS Kunkel D Petitjean G Dillies MA Roques P Butor C Silvestri G Giavedoni LD Lebon P Barré-Sinoussi F Benecke A Müller-Trutwin MC Nonpathogenic SIV infection of African green monkeys induces a strong but rapidly controlled type I IFN response J Clin Invest 2009 119 3544 3555 19959873 10.1172/JCI40093 PMC2786805

[B40] Grossman Z Meier-Schellersheim M Paul WE Picker LJ Pathogenesis of HIV infection: what the virus spares is as important as what it destroys Nat Med 2006 12 289 295 10.1038/nm1380 16520776

[B41] Ferbas J Navratil J Logar A Rinaldo C Selective decrease in human immunodeficiency virus type 1 (HIV-1)-induced alpha interferon production by peripheral blood mononuclear cells during HIV-1 infection Clin Diagn Lab Immunol 1995 2 138 142 7697520 10.1128/cdli.2.2.138-142.1995 PMC170116

[B42] Kamga I Kahi S Develioglu L Lichtner M Marañón C Deveau C Meyer L Goujard C Lebon P Sinet M Hosmalin A Type I interferon production is profoundly and transiently impaired in primary HIV-1 infection J Infect Dis 2005 192 303 310 10.1086/430931 15962225

[B43] Ito T Kanzler H Duramad O Cao W Liu YJ Specialization, kinetics, and repertoire of type 1 interferon responses by human plasmacytoid predendritic cells Blood 2006 107 2423 2431 10.1182/blood-2005-07-2709 16293610

[B44] Tilton JC Manion MM Luskin MR Johnson AJ Patamawenu AA Hallahan CW Cogliano-Shutta NA Mican JM Davey RT Jr Kottilil S Lifson JD Metcalf JA Lempicki RA Connors M Human immunodeficiency virus viremia induces plasmacytoid dendritic cell activation in vivo and diminished alpha interferon production in vitro J Virol 2008 82 3997 4006 10.1128/JVI.01545-07 18256146 PMC2293017

[B45] Khatissian E Tovey MG Cumont MC Monceaux V Lebon P Montagnier L Hurtrel B Chakrabarti L The relationship between the interferon alpha response and viral burden in primary SIV infection AIDS Res Hum Retroviruses 1996 12 1273 1278 10.1089/aid.1996.12.1273 8870849

[B46] Fonteneau JF Larsson M Beignon AS McKenna K Dasilva I Amara A Liu YJ Lifson JD Littman DR Bhardwaj N Human immunodeficiency virus type 1 activates plasmacytoid dendritic cells and concomitantly induces the bystander maturation of myeloid dendritic cells J Virol 2004 78 5223 5232 10.1128/JVI.78.10.5223-5232.2004 15113904 PMC400371

